# Identification of the Key Genes and Potential Therapeutic Compounds for Abdominal Aortic Aneurysm Based on a Weighted Correlation Network Analysis

**DOI:** 10.3390/biomedicines10051052

**Published:** 2022-05-02

**Authors:** Lin Li, Kejia Kan, Prama Pallavi, Michael Keese

**Affiliations:** 1 Department of Vascular Surgery, Medical Faculty Mannheim, Heidelberg University, 68167 Mannheim, Germany; lin.li@medma.uni-heidelberg.de (L.L.); kejia.kan@medma.uni-heidelberg.de (K.K.); prama.pallavi@medma.uni-heidelberg.de (P.P.); 2 European Center for Angioscience ECAS, Medical Faculty Mannheim, Heidelberg University, 68167 Mannheim, Germany

**Keywords:** AAA, WGCNA, CTD

## Abstract

Background: There is still an unmet need for therapeutic drugs for patients with an abdominal aortic aneurysm (AAA), especially for candidates unsuitable for surgical or interventional repair. Therefore, the purpose of this in silico study is to identify significant genes and regulatory mechanisms in AAA patients to predicate the potential therapeutic compounds for significant genes. Methods: The GSE57691 dataset was obtained from Gene Expression Omnibus (GEO) and used to identify the differentially expressed genes (DEGs) and weighted correlation network analysis (WGCNA). The biological function of DEGs was determined using gene ontology (GO) and the Kyoto Encyclopedia of Genes and Genomes (KEGG). AAA-related genes were obtained from the Comparative Toxicogenomics Database (CTD) using the keywords: aortic aneurysm and abdominal. The hub genes in AAA were obtained by overlapping DEGs, WGCNA-based hub genes, and CTD-based genes. The diagnostic values of hub genes were determined using ROC curve analysis. Hereby, a TF-miRNA-hub gene network was constructed based on the miRnet database. Using these data, potential therapeutic compounds for the therapy of AAA were predicted based on the Drug Gene Interaction Database (DGIdb). Results: A total of 218 DEGs (17 upregulated and 201 downregulated) and their biological function were explored; 4093 AAA-related genes were derived by text mining. Three hub modules and 144 hub genes were identified by WGCNA. asparagine synthetase (ASNS), axin-related protein 2 (AXIN2), melanoma cell adhesion molecule (MCAM), and the testis-specific Y-encoded-like protein 1 (TSPYL1) were obtained as intersecting hub genes and the diagnostic values were confirmed with ROC curves. As potential compounds targeting the hub genes, asparaginase was identified as the target compound for ASNS. Prednisolone and abiraterone were identified as compounds targeting TSPYL1. For MCAM and TSPYL1, no potential therapeutic compound could be predicted. Conclusion: Using WGCNA analysis and text mining, pre-existing gene expression data were used to provide novel insight into potential AAA-related protein targets. For two of these targets, compounds could be predicted.

## 1. Introduction

Abdominal aortic aneurysm (AAA) is a common and potentially life-threatening disease that leads to more than 150,000 global deaths yearly [1,2,3,4,5]. While there is conclusive data on the role of environmental factors in the development of AAA in patients, there are also distinct genetic factors that have been found to play an important role in AAA progression [6]. These large dataset-based RNA-seq and microarrays datasets include differentially expressed single genes (for example, H19, BRG1-associated factor 60A (BAF60a), Kruppel-like factor 5 (Klf5), and thyroid receptor-interacting protein 13 (TRIP13)) [7,8,9], single nucleotide polymorphism (SNP) of genes such as the matrix metalloproteinase (MMP) family, transforming growth factor-beta (TGFBR2), and sortilin (SORT1) [10], or gene families such as the MMP family.

MicroRNAs (miRNAs) are a class of non-coding RNAs that regulate target gene expression by region binding to the 3’ UTR of target mRNA and regulation of many bioprocesses to silence and activate the target gene expression at the transcriptional and post-transcriptional level [11,12,13]. These oligonucleotides have also been associated with the development of AAA [14]. Interestingly, a large number of miRNAs have been shown to be associated with the regulation of key gene expression during the pathophysiology of AAA. One example of these is miRNA-21 (miR-21). Overexpression of miR-21 can inhibit the viability of vascular smooth muscle cells (VSMCs) and stabilize the aortic wall in animal models of AAA by downregulating the expression of phosphatase and tensin homolog (PTEN) [15].

MiRNAs may also interact with other key players for the development of AAA: transcription factors (TFs). By interacting with miRNAs, TFs act as master regulators of several genes at once by constituting transcriptional complexes. Identifying miRNAs and their respective TFs from co-expressed genes can thus provide important insights into the regulatory mechanism affecting vascular disorders such as AAA [14,16]. During AAA progression, miRNAs have been shown to regulate the extracellular matrix (ECM) turnover, the matrix metalloproteinase (MMP) family, different inflammatory components, and VSMCs by forming TF–miRNA network networks [17].

Based on the public dataset and bioinformatic analysis, multiple studies have been conducted to explore the regulatory mechanism of AAA at the genetic level [18,19,20,21]. Public datasets for further analysis are still scarce. In our study, we combined the GEO dataset with the Comparative Toxicogenomics Database (CTD) dataset to identify differential genes and reveal potential TF–miRNA-hub gene regulatory networks related to AAA.

## 2. Materials and Methods

### 2.1. Data Download and Processing

**Figure 1:**
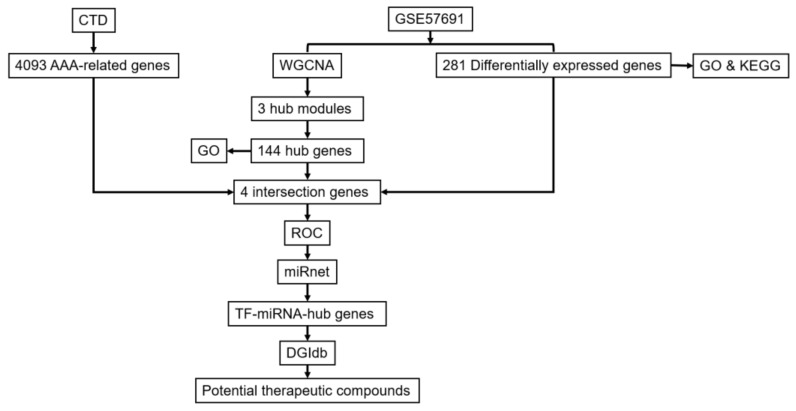
Flowchart of the data analysis.

Microarray data of the GSE57691 dataset, which includes 49 AAA samples (20 patients with small AAA (≤55 mm), 29 patients with large AAA (>55 mm)), and 10 normal control full-thickness aortic wall biopsies and corresponding clinical data were downloaded from gene expression omnibus (GEO, https://www.ncbi.nlm.nih.gov/geo/, 23 August 2021) [22]. The expression data were generated by Illumina HumanHT-12 V4.0 expression bead chips [22]. In the present study, the normalized data were downloaded and used for subsequent analyses. Figure 1 displays the flow chart of the data analysis.

### 2.2. AAA-Related Genes Obtained from Comparative Toxicogenomics Database (CTD)

The Comparative Toxicogenomics Database (CTD: http://ctdbase.org, 23 August 2021) is a public database that contains a wide spectrum of information on chemicals, genes, phenotypes, diseases, and exposures to advance the understanding of human health [11,18]. A total of 21,684 AAA-related genes were obtained from CTD using the keywords aortic aneurysm and abdominal and 4093 AAA-related genes were found according to previous studies (Table S5) [19,20].

### 2.3. Identification of the Differentially Expressed Genes (DEGs) in AAA and Normal Tissues

The DEGs between AAA and normal tissues were screened by the Limma R 3.46.0 package [22] with cutoff values of |log2 (fold change, FC) | > 1 and *p*-value < 0.05. All results were visualized by the ggplot2 R 3.3.3 package [22].

### 2.4. Gene Ontology (GO) Annotation and Kyoto Encyclopedia of Genes and Genomes (KEGG) Analysis

Gene ontology (GO) annotation and Kyoto Encyclopedia of Genes and Genomes (KEGG) pathway enrichment analysis, GO annotation including biological process (BP), cellular component (CC), molecular function (MF), and KEGG pathway enrichment analyses were performed by the cluster profile R 3.14.3 package [23] with false discovery rate (FDR) < 0.05 and the analyses were visualized by the ggplot2 R 3.3.3 package.

### 2.5. Weighted Gene Co-Expression Network Analysis (WGCNA)

The transcripts and samples from the GSE57691 dataset were analyzed by the WGCNA R 1.70-3 package [22]. All samples were clustered according to Pearson’s correlation analysis and the outliers were removed. The community dissimilarity was speculated according to the similarity. Afterward, the adjacency matrix was transformed into a topological overlap matrix (TOM). Genes were assigned to different gene modules according to the TOM-related dissimilarity measure and the soft-thresholding setting. The numbers of gene modules were obtained according to the dissimilarity and the criterion of dynamic tree cutting with a minimal module size of 30 genes. The modules that correlated the most with the clinical traits were identified as AAA-related modules in this study. All biological functions of the hub genes with gene significance (GS) > 0.2 and module membership (MM) > 0.8 were analyzed by GO enrichment.

### 2.6. Identification of the Hub Genes in AAA Based on Receiver Operating Characteristic (ROC) Curve Analysis

Two-hundred and eighteen DEGs between AAA and normal tissues, 4093 AAA-related genes from CTD, and 144 hub genes from WGCNA were overlapped to identify the intersection genes in AAA. The diagnostic values of four intersection genes for AAA were detected by ROC curve analysis and the area under the ROC curve (AUC) using the pROC R 1.17.0.1 package [24].

### 2.7. Construction of the Transcription Factor (TF)-miRNA-Hub Gene Network

MiRNAs and TFs related to four hub genes were screened out based on the miRNet2/0 online database (https://www.mirnet.ca, 23 August 2021) [18]. Nine TFs and 290 miRN0As related to four hub genes were identified and constructed in the network using Cytoscape (San Diego, CA, USA) [24].

### 2.8. Screening the Potential Therapeutic Compounds for AAA in Drug-Gene Interaction Database (DGIdb)

DGIdb (https://www.dgidb.org, 23 August 2021) was used as a drug–gene interaction database to supply the drug–gene interactions and gene–drug ability information from papers, databases, and web resources [24]. In the present study, the target therapeutic compounds for four hub genes were identified based on the DGIdb.

## 3. Results

### 3.1. Identification of the DEGs and Analysis of Their Function in AAA

**Figure 2:**
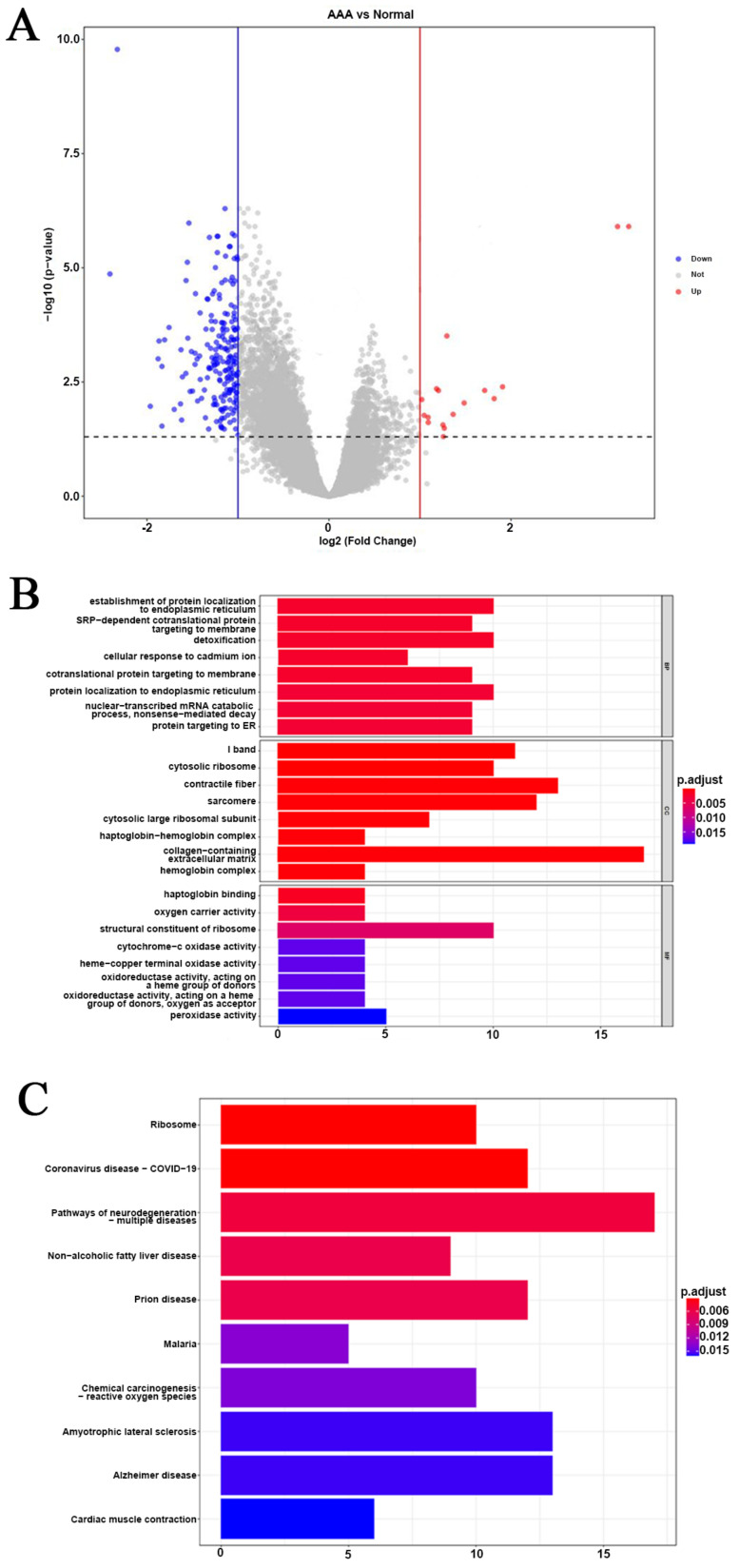
Identification of DEGs and analysis of their function in AAA. (**A**) Volcano plot of DEGs with cutoff value |log2 (FC)| > 1 and *p*-values < 0.05. (**B**) Bar charts of the GO analysis with FDR > 0.05, including BP, CC, and MF terms. (**C**) Bar charts of the KEGG pathway enrichment analysis with FDR > 0.05.

A total of 218 DEGs, including 17 upregulated and 201 downregulated DEGs, were identified from the GSE57691 dataset using the Limma R package [22,23,24] with the thresholds |log2 (FC) | > 1 and *p*-value < 0.05 (Figure 2A and Table S1). The biological function of these genes was explored by GO and KEGG enrichment analyses with FDR < 0.05. The most significant BP terms included establishment of protein localization to endoplasmic reticulum, signal recognition particle (SRP)-dependent co-translational protein targeting to membrane, detoxification, cellular response to cadmium ion, and co-translational protein targeting to membrane (Figure 2B and Table S2). The significant CC terms included I band, cytosolic ribosome, contractile fiber, sarcomere, and cytosolic large ribosomal subunit. The significant MF terms included haptoglobin binding, oxygen carrier activity, structural constituent of ribosome, cytochrome-c oxidase activity, and heme-copper terminal oxidase activity. Several genes were also significantly associated with several degenerative disease-related pathways and others, such as ribosome, cardiac muscle contraction, oxidative phosphorylation, fatty acid degradation, pyruvate metabolism, and diabetic cardiomyopathy based on KEGG (Figure 2C and Table S3).

### 3.2. Identification of the Hub Modules and Genes by WGCNA

**Figure 3:**
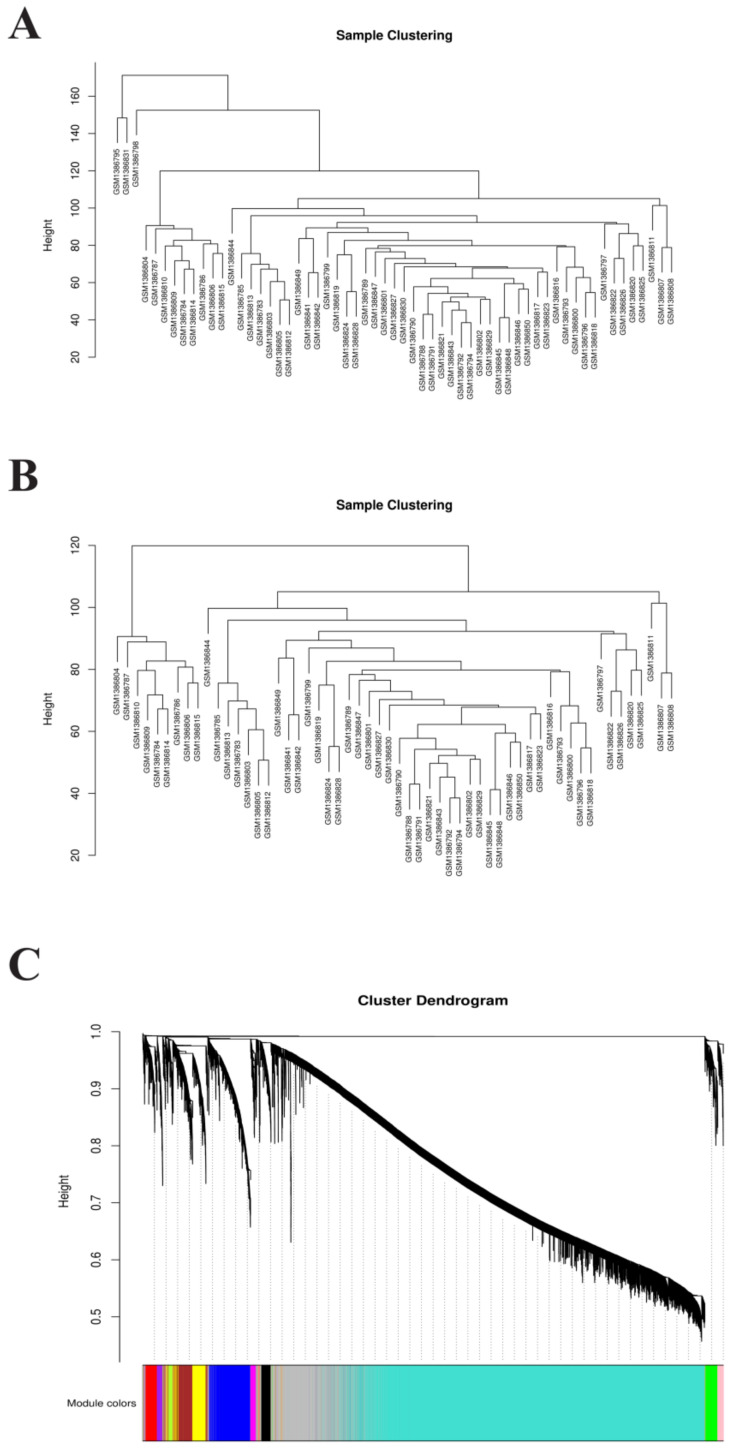
Soft-setting of threshold power. (**A**,**B**) GSM1386795, GSM1386831, and GSM1386798 were excluded as outliers. (**C**) The minimum number of genes per module was set to 30 according to the criteria of the dynamic tree-cutting algorithm. Thirteen modules were generated. Genes are grouped into modules by hierarchical clustering, with different colors representing different modules, where the grey default is for genes that cannot be grouped into any module.

**Figure 4:**
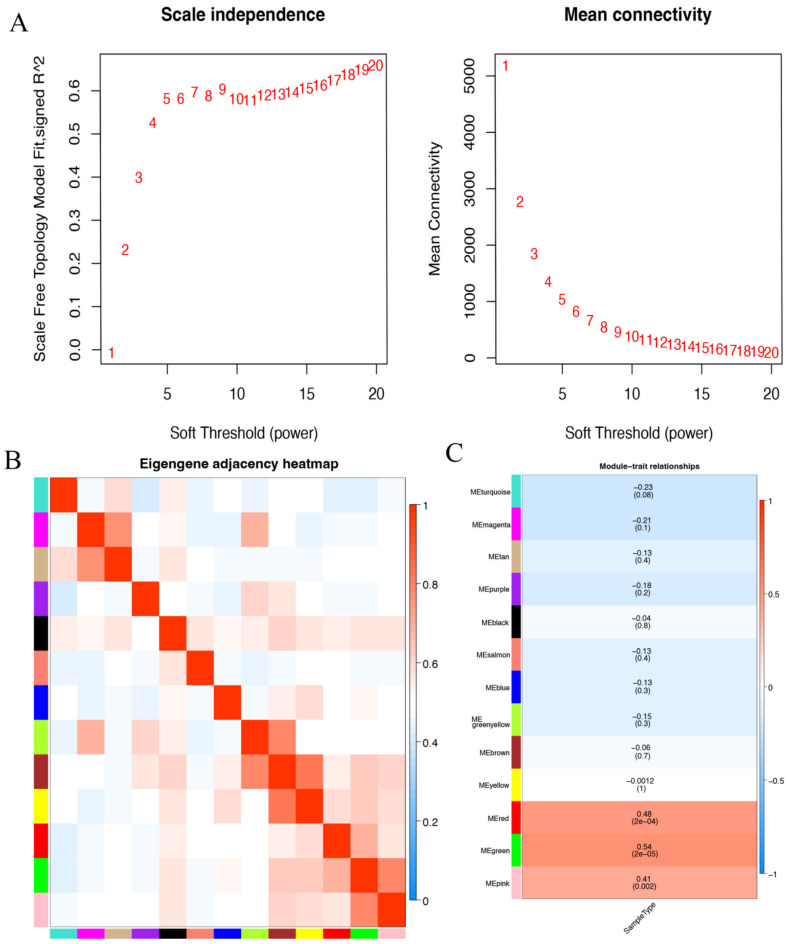

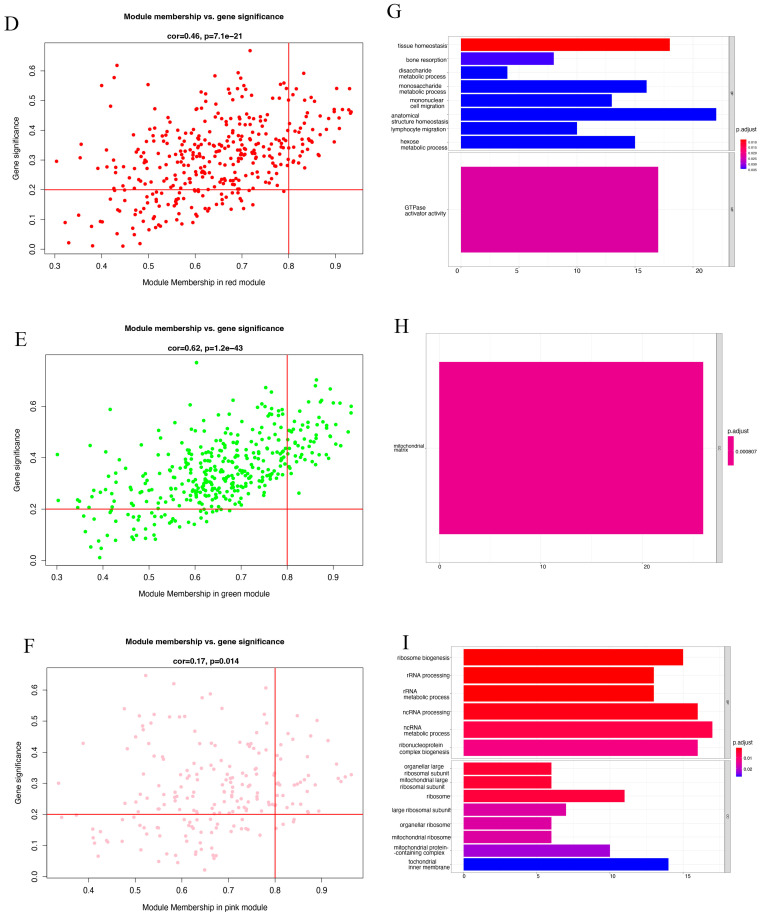
Identification of the hub modules and genes by WGCNA. (**A**) The soft-threshold power selecting processes included the scale-free fit index (left) and the mean connectivity (right) analysis. (**B**) Eigengene adjacency heatmap of correlation between the modules. (**C**) Heatmap of the correlation between module eigengenes and clinical traits of AAA. (**D**–**F**) Scatter plots of the correlation between MM and GS of red, green, and pink modules, respectively. (**G**–**I**) Bar charts of the GO analysis of genes from red, green, and pink modules, respectively.

A total of 17,784 genes were derived from the 49 samples from the GSE57691 dataset. These genes were used to construct the co-expression network. The results of the cluster analysis of the samples are shown in Figure 3 and Figure 4A,B. All outliers, including GSM1386795, GSM1386831, and GSM1386798 were removed and the remaining 46 samples were used for subsequent analysis (Figure 3A,B). Because the scale-free topology fit index (signed R^2^) was less than 0.85 (Figure 4A), a soft-setting of threshold power was achieved according to the criterion with samples > 40 (Figure 3A). When the soft-threshold power was set as 12 and 13, modules could be identified with a minimal module size of 30 genes (Figure 4B).

To correlate the modules with sample information, we analyzed the data according to the heatmap of module–clinical trait correlations. Hereby, we correlated data for the clinical traits. Hereby, red, green, and pink modules were identified as the most correlated with clinical traits (Figure 4C). The red and green ones, which were identified as the hub modules associated with clinical traits, were used to deeply explore the correlation between module membership (MM) and gene significance (GS) to identify the hub genes in AAA. In the results demonstrated in Figure 4D–F and Table S4, 47, 60, and 37 hub genes were respectively identified from red, green, and pink modules with the MM > 0.8 and GS > 0.2. Furthermore, the biological function of the hub genes from three modules was analyzed by GO analysis with FDR < 0.05. The results revealed that the hub genes of the red module are mostly enriched in tissue homeostasis, bone resorption, disaccharide metabolic process, monosaccharide metabolic process, mononuclear cell migration, anatomical structure homeostasis, lymphocyte migration, and hexose metabolic process. All genes are associated with GTPase activator activity (Figure 4G). The hub genes of the green module significantly correlated with mitochondrial matrix (Figure 4H). The hub genes of the pink module are enriched in ribosome biogenesis, rRNA processing, rRNA metabolism process, ncRNA processing, ncRNA metabolism processing, and ribonucleoprotein complex biogenesis. The CC terms included organellar, large ribosomal subunit, mitochondrial large ribosomal subunit, ribosome, large ribosome subunit, organellar ribosome, mitochondrial ribosome, mitochondrial protein-containing complex, and mitochondrial inner membrane (Figure 4I).

### 3.3. Identification and Validation of AAA-Related Hub Genes

**Figure 5:**
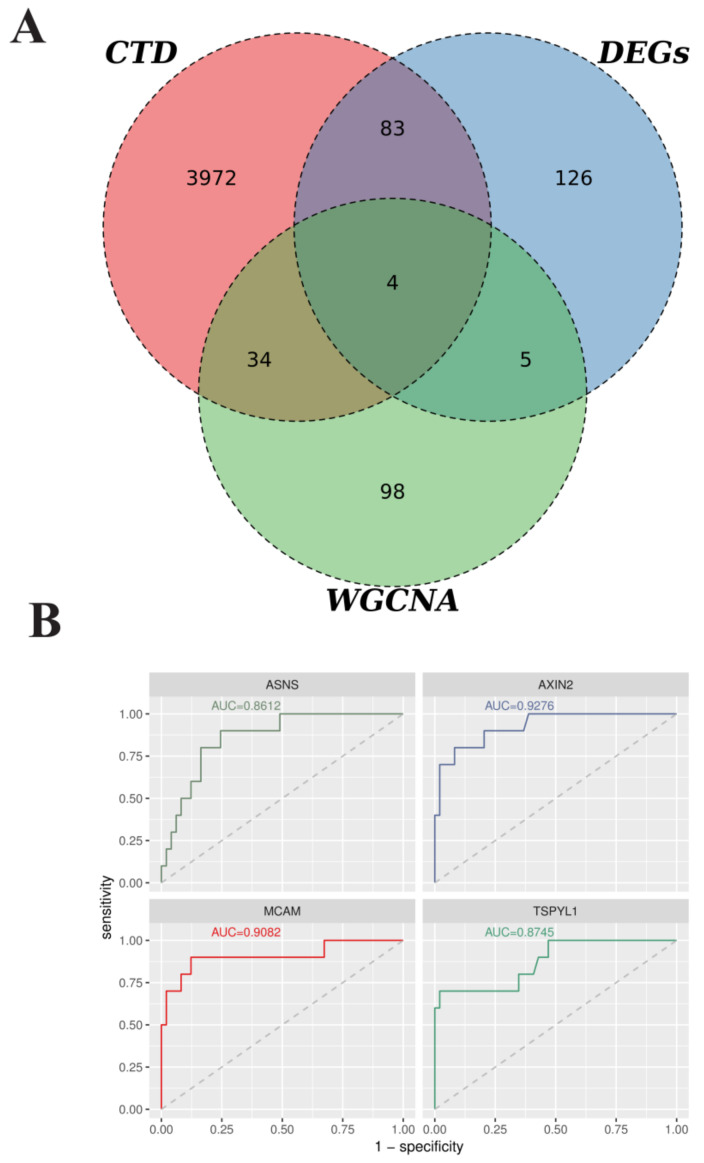
Identification and validation of the AAA-related hub genes. (**A**) Venn plots of intersection genes among the AAA-related 218 DEGs, 144 WGCNA-based hub genes, and 4093 CTD-based genes. (**B**) ROC curves and AUC values of the ASNS, AXIN2, MCAM, and TSPYL1. x-axis indicates specificity; y-axis indicates sensitivity.

Based on CTD (search keywords: aortic aneurysm and abdominal), a total of 21,684 AAA-related genes were obtained. Moreover, 4093 AAA-related genes included genes with more than 4 references (Table S2). By overlapping the AAA-related 218 DEGs, the 144 WGCNA-based hub genes, and the 4093 CTD-based genes, 4 AAA-related genes were obtained (Figure 5A). Then, the diagnostic values of four genes for AAA were confirmed by ROC curve analysis and the AUC value. As shown in Figure 5B, the AUC values of ASNS, AXIN2, MCAM, and TSPYL1 were shown to be of prognostic power in AAA with 0.8612, 0.9276, 0.9082, 0.8745, respectively.

### 3.4. Construction of the TF-miRNA-Hub Gene Network in AAA

**Figure 6:**
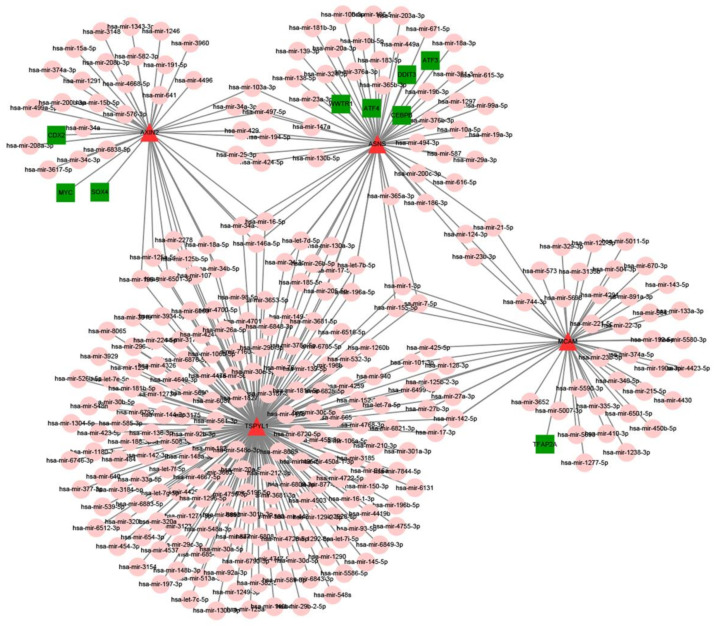
Construction of the TF–miRNA-hub gene network in AAA based on miRnet. Red triangle indicates 4 hub genes (ASNS, AXIN2, MCAM, TSPYL1), pink circles display miRNAs, and green squares show TFs.

We further investigated the regulatory mechanism of these four genes in AAA. The target miRNAs and TFs of four genes were identified and then the TF–miRNA-hub gene network was constructed based on miRnet. Finally, a TF–miRNA-hub gene network, which included 4 genes, 9 TFs, and 290 miRNAs, was constructed with 347 edges (Figure 6).

### 3.5. Identification of the Potential Therapeutic Compounds for AAA

In the search for common potential therapeutic compounds for the four hub genes, DGIdb was used (Table 1). Asparaginase has been identified as the target compound of ASNS; prednisolone and abiraterone were found as the target compounds of TSPYL1. For the other two genes, no drug compound could be identified.

## 4. Discussion

To date, several druggable molecules have been described to play roles in AAA development, progression, and rupture, such as the matrix metalloproteinase (MMP) family, transforming growth factor beta receptor 2 (TGFBR2), and sortilin 1 (SORT1) [10]. However, not only single protein-expressing genes may be involved but also whole pathway networks may be deregulated, such as the Wnt/β-catenin pathway and TGF beta/SMAD pathway. This may be mediated by miRNAs. MiRNAs have been studied to be associated with the pathophysiological process of many diseases [11]. Various studies have reported that miRNAs are involved in the occurrence and development of AAA [12]. Especially, it is important to construct TF–miRNA-hub gene networks for miRNAs as they may regulate several pathways. These networks may help to find novel therapeutic compounds for significant genes.

Here, we built an in silico approach TF–miRNA-hub gene network depending on the shared dataset and published literature. Unlike our previous study which only focused on WCGNA to mine key genes of mouse AAA progression [21], we here applied text mining and WCGNA. Hereby, four hub genes (ASNS, AXIN2, MCAM, TSPYL1) were obtained. These four hub genes are all reported to be associated with the function of vascular cells, but no study has so far studied the connection between these four hub genes and the pathophysiology of AAA [23,24].

Asparagine synthetase (ASNS) is associated with asparaginase therapy in acute lymphoblastic leukemia [25]. ASNS is also reported to be essential for endothelial cell growth and angiogenesis [26]. Interestingly, the proliferation of fibroblasts is impaired under conditions of asparagine deprivation which may be the potential link connecting ASNS and AAA pathogenesis [26].

Testis-specific protein, Y-encoded-like 1 (TSPYL1) is a member of the TSPYL protein family which exerts its regulatory effects on vascular cells and promotes endothelial cell proliferation, migration, and neoangiogenesis [27]. AXIN2, a scaffold protein involved in the degradation of β-catenin, plays a vital role in the Wnt/β-catenin pathway and its gene expression is suggested to be associated with endothelial cell proliferation and function [28]. AXIN2 mutations have been studied in different cancer entities including digestive tract tumors and melanoma [29]. MCAM is highly expressed in many tumors and endothelial cells. It plays an important role in the regulation of vascular permeability, cell–cell cohesion, leukocyte transmigration, and angiogenesis [30].

MiRNAs may be a potential tie between TSPYL5, AXIN2, and MCAM and AAA formation. MiRNAs have been shown to regulate the ECM turnover, MMP family, different inflammatory components, and VSMCs by forming TF–miRNA network networks [17].

We here identified nine TFs and 290 miRNAs as the master regulators of the resulting gene regulatory network, as they have the largest connectivity with the co-expressed four genes associated with AAA. The diagnostic values of the four genes for AAA were confirmed using ROC curves. These data confirmed that ASNS, AXIN2, MCAM, and TSPYL1 showed significant prognostic values in AAA.

The compound search was based on the analysis of a TF–miRNA-hub gene network. Increasing evidence has indicated that TF-related networks exert key roles in cancer. A prominent example is the SOX4–Axin2 network and the CDX2–Axin2 network, both of which are reported to inhibit the proliferation and tumor formation of cancer cells by suppressing Wnt/β-catenin signaling [31]. The WWTR1–ASNS network, ATF4–ASNS network, and DDIT3–ASNS network are reported to inhibit the proliferation and tumor formation of cancer cells [32]. The CEBPB–ASNS network and ATF3–ASNS network can activate the placental mammalian amino acid response pathway [33]. Furthermore, the TFAP2A–MCAM network is reported to be associated with melanoma metastasis [34]. All TF–miRNA networks have been previously associated with the development of AAA.

With the help of DGIdb, the target therapeutic compounds for two of the hub genes were identified. Asparaginase has been identified as the target compound of ASNS, while prednisolone and abiraterone were identified as target compounds of TSPYL1. Since all four hub genes were firstly connected to AAA in the present study, there is so far no experimental evidence for the drug compounds. This will also be an issue for further experimental studies.

In summary, our study has firstly demonstrated a novel TF–miRNA-hub network linked to AAA based on text mining and WCGNA analysis. While this is a novel approach with novel findings, our work also has some limitations. Our work has focused on potential regulators of the most significantly overexpressed genes, TFs, and miRNAs of AAA, without ruling out the possibility that other regulatory mechanisms, which may not only depend on gene overexpression, may still be important in the development and progression of AAA. Therefore, our in silico findings will have to be confirmed by in vitro and in vivo AAA models.

Moreover, the number of datasets available is still limited. We here used the GSE57691 dataset as the most complete available dataset to combine hits with the AAA-related genes that we obtained from CTD to identify the DEGs.

## Figures and Tables

**Table 1:** The potential compounds of two genes were identified using DGIdb.

Gene	Drug	Interaction Type	Sources	PMIDs
ASNS	ASPARAGINASE	NASE	NCI, CIViC, PharmGKB	28069604, 24268318, 11556848
TSPYL1	PREDNISOLONE	LONE	PharmGKB	
TSPYL1	ABIRATERONE	ONE	PharmGKB	

## Data Availability

All datasets of this study are available in the GEO database (https://www.ncbi.nlm.nih.gov/geo/, accessed on 23 August 2021).

## Supplementary Materials

The following supporting information can be downloaded at: https://www.mdpi.com/article/10.3390/biomedicines10051052/s1, Table S1: Differentially expressed gene; Table S2: GO analysis; Table S3: KEGG analysis; Table S4: hub genes of hub modules; Table S5: AAA-related genes from CTD.
