# Supplementary figures and images for: Identification of the Key Genes and Potential Therapeutic Compounds for Abdominal Aortic Aneurysm Based on a Weighted Correlation Network Analysis
Source: Biomedicines. 2022 May 2;10(5):1052. doi: 10.3390/biomedicines10051052 (PMC9138830; doi:10.3390/biomedicines10051052)

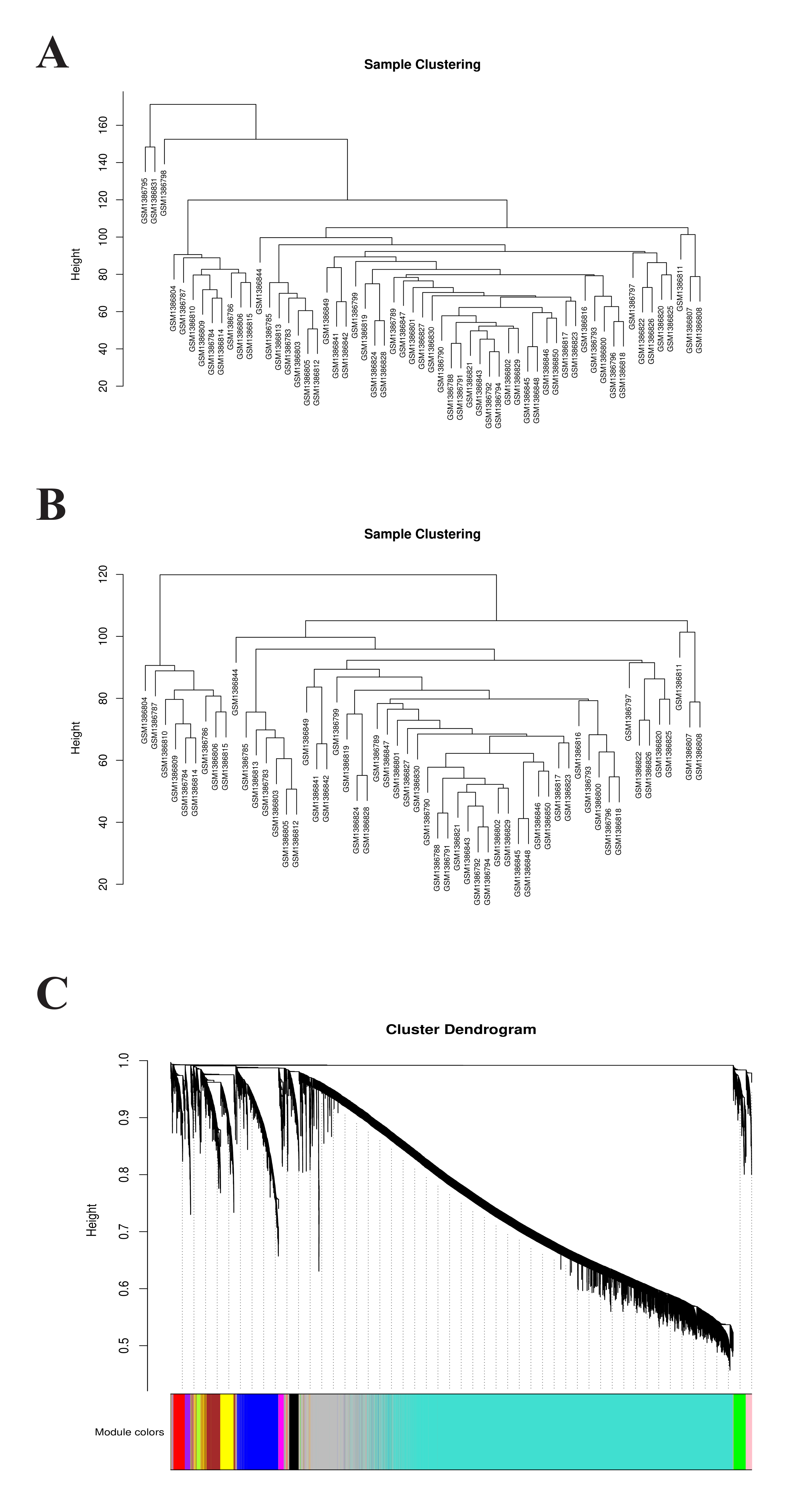

Supplement: Supplementary file 1 [file biomedicines-10-01052-s001.zip › biomedicines-1712327-supplementary/supplement/Supplemtn Figure.tif]
